# Control of phosphodiesterase activity in the regulator of biofilm dispersal RbdA from *Pseudomonas aeruginosa*†

**DOI:** 10.1039/d4cb00113c

**Published:** 2024-08-27

**Authors:** Charlotte Cordery, Jack Craddock, Martin Malý, Kieran Basavaraja, Jeremy S. Webb, Martin A. Walsh, Ivo Tews

**Affiliations:** a Biological Sciences, Institute for Life Sciences, University of Southampton Southampton SO17 1BJ UK ivo.tews@soton.ac.uk; b National Biofilms Innovation Centre, University of Southampton Southampton SO17 1BJ UK; c Diamond Light Source, Harwell Science and Innovation Campus Didcot Oxfordshire OX11 0DE UK; d Research Complex at Harwell, Harwell Science and Innovation Campus Didcot Oxfordshire OX11 0FA UK

## Abstract

The switch between planktonic and biofilm lifestyle correlates with intracellular concentration of the second messenger bis-(3′-5′)-cyclic dimeric guanosine monophosphate (c-di-GMP). While bacteria possess cyclase and phosphodiesterase enzymes to catalyse formation or hydrolysis of c-di-GMP, both enzymatic domains often occur in a single protein. It is tacitly assumed that one of the two enzymatic activities is dominant, and that additional domains and protein interactions enable responses to environmental conditions and control activity. Here we report the structure of the phosphodiesterase domain of the membrane protein RbdA (regulator of biofilm dispersal) in a dimeric, activated state and show that phosphodiesterase activity is controlled by the linked cyclase. The phosphodiesterase region around helices α5/α6 forms the dimer interface, providing a rationale for activation, as this region was seen in contact with the cyclase domain in an auto-inhibited structure previously described. Kinetic analysis supports this model, as the activity of the phosphodiesterase alone is lower when linked to the cyclase. Analysis of a computed model of the RbdA periplasmatic domain reveals an all-helical architecture with a large binding pocket that could accommodate putative ligands. Unravelling the regulatory circuits in multi-domain phosphodiesterases like RbdA is important to develop strategies to manipulate or disperse bacterial biofilms.

## Abstract 2

The structural study of RbdA shows an activated phosphodiesterase, required for bacterial biofilm dispersal.

## Graphical abstract

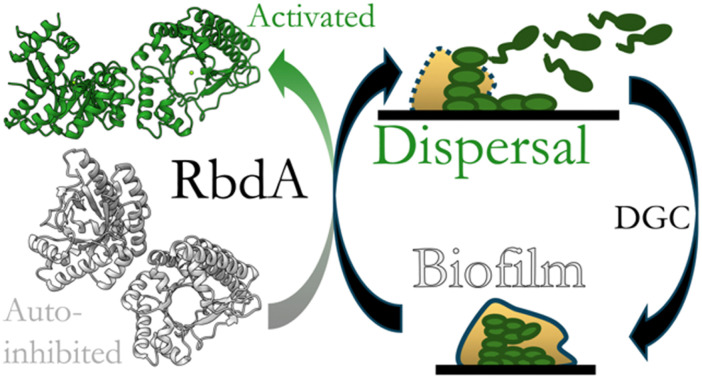

## Introduction

*Pseudomonas aeruginosa* is a rod-shaped aerobic and facultative anaerobic Gram-negative bacterium ubiquitous in the environment. As an opportunistic biofilm-forming pathogen, *P. aeruginosa* can cause complications across a wide range of disease areas,^1^ exacerbated by the prevalence of antibiotic resistance and by the propensity of the organism to form biofilms.^2,3^ As one example, *P. aeruginosa* accounts for the highest rate of morbidity and health in cystic fibrosis patients through chronic infection and inflammation of the lung, resulting in tissue damage that is ultimately fatal.^4,5^ Despite the discovery of CFTR modulator therapies,^6,7^*P. aeruginosa* continues to cause chronic infections, threatening healthcare in cystic fibrosis.^8^

Physiological changes between planktonic and biofilm lifestyle correlate with changes in the concentration of the secondary messenger bis-(3′-5′)-cyclic dimeric guanosine monophosphate (c-di-GMP).^9^ The biofilm state is associated with high intracellular c-di-GMP concentration; synthesis of c-di-GMP from guanosine triphosphate (GTP) by diguanylate cyclase enzymes (DGCs) thus controls biofilm formation.^10^ In contrast, c-di-GMP hydrolysis is catalysed by phosphodiesterase enzymes (PDEs) to control dispersal.^11^ Tandem domain architectures coupling DGCs and PDEs are common amongst biofilm regulatory proteins.^12^ This architecture is puzzling as the presence of catalytic domains with opposing activity poses questions to regulation and balancing of activities.

We studied the regulator of biofilm dispersal (RbdA). RbdA is a membrane protein, containing two transmembrane regions with an interspersed periplasmatic domain of uncharacterised function. The cytosolic portion of RbdA contains a PAS domain (Per-Arnt-Sim). PAS domains are frequently found amongst bacterial sensory systems^13,14^ and play crucial roles in environmental responses.^15,16^ They are also widespread in c-di-GMP regulatory enzymes where they are suggested to play a role in the regulation of virulence,^17^ as well as in motility and biofilm phenotype.^18^ The PAS domain is followed by a DGC–PDE dimer. Dominance of phosphodiesterase activity in RbdA was demonstrated by replacement of the conserved “EAL” signature motif that is required for activity, resulting in increased biofilm formation. Thus, the RbdA PDE activity was suggested to down-regulate biofilm formation under normal circumstances.^19^

RbdA catalyses hydrolysis of c-di-GMP to pGpG as previously shown by high-performance liquid chromatography analysis.^20^ Using a Δ*rbdA* deletion mutant, we reported several physiological changes compared to wild-type PAO1 that can be associated with dispersal. These include higher c-di-GMP levels, thicker biofilms, a five-fold increase in microcolony size, and a two-fold increase in biomass after 48 h with enhanced total protein and polysaccharide.^18^ The RbdA protein was required for NO-induced biofilm dispersal, consistent with reduced swimming and swarming motility in the Δ*rbdA* deletion mutant following NO donor addition.^18^ The *P. aeruginosa* Δ*rbdA* deletion mutant also failed to respond to dispersal signals such as glutamate, mercury chloride, or ammonium chloride.^21^

The interfacing area required for PDE dimer formation and activation was buried in the previously characterised autoinhibited state of the PAS/DGC/PDE fragment,^19^ we provide kinetic data to confirm that the DGC domain alone can exert an inhibitory effect on PDE activity. A computed structure approach was used to describe the periplasmatic domain of hitherto unknown function, analysis of which suggests ligand binding in a cavity that fits ligands of up to ∼600 Da.

## Experimental

### Molecular biology

Genome extraction from *P. aeruginosa* strain PAO1 overnight cultures was performed using the Wizard genomic DNA purification kit (Promega), with incubation at 37 °C for 30 min, DNA pellet rehydration with 200 μL of rehydration solution, and all centrifugation steps were carried out at 16 000 × *g*. The following primers were used to generate RbdA-DGC (RbdA_376–536_), RbdA-PDE (RbdA_549–797_) and RbdA-DGC–PDE (RbdA_376–797_): RbdA376FWD aactt**CATATG**CACGATGCGTTGACCG, RbdA536REV aactt**AAGCTT**TCAATGGAACACCTGGACCCG, RbdA549FWD aacttCATATGACCTGGGTCCAGCG, RbdA797REV aactt**AAGCTT**TCAGCGACTGAACGGCAGG (restriction sites bold, excess sequence in small letters). PCR amplification used PCR Phusion or Q5 high-fidelity DNA Polymerase (NEB). PCR products were purified using the QIAquick PCR Purification Kit (QIAGEN). Cloning into pET28a (Novagen) used NdeI/HindIII restriction, dephosphorylation with shrimp alkaline phosphatase, and T4 DNA ligase (all NEB). The periplasmic RbdA_38–201_ used a synthetic gene with N-terminal signal sequence QRRYTMKIKTGARILALSALTTMMFSASALAA and C-terminal His-tag (Eurofins), inserted into pOPINF vector using the PCR amplification and In-Fusion HD EcoDry mix (Takara Bio).

### Protein expression and purification

Overexpression of cytoplasmic domains used *E. coli* BL21 (DE3) (NEB) cells transformed with the respective plasmids in Lysogeny Broth (LB) with added selective antibiotic (50 μg mL^−1^ kanamycin). Cultures were set up at 37 °C in 2 L baffled flasks under 180 rpm shaking. At OD_600_ ∼0.2, the temperature was reduced to 18 °C, and growth continued for 18 h after induction at OD_600_ ∼0.6 with 1 mM final concentration IPTG (Fisher Scientific). Overexpression of the periplasmic domain used *E. coli* Rosetta cells grown in Autoinduction Media Terrific Broth (Formedium) with added selective antibiotic (100 μg mL^−1^ carbenicillin and 34 μg mL^−1^ chloramphenicol). Cells were grown at 37 °C for 6 hours, reducing the temperature to 18 °C, and grown for 16–72 hours. Cells were harvested by centrifugation and shock-frozen until use. For purification of the cytoplasmic domains, cells were resuspended in lysis buffer (50 mM Tris, 200 mM NaCl, 5% (v/v) glycerol, pH 7.5 or pH 8.0, 2 mM β-mercaptoethanol), sonicated, and the soluble fraction harvested by centrifugation (92 600 × *g*, 4 °C, 40 min). Purification used Ni-NTA Superflow resin (Qiagen) in a gravity flow column pre-equilibrated with lysis buffer containing 20 mM imidazole. After washing with lysis buffer containing 80 mM imidazole, proteins were eluted with lysis buffer containing 300 mM imidazole and 300 mM NaCl. Following concentration on a 10 000 MWCO Vivaspin 20 (Sartorius), the eluate was loaded onto a HiLoad 16/600 Superdex 75 size exclusion column (GE Healthcare) pre-equilibrated with gel filtration buffer (50 mM Tris, 300 mM NaCl, pH 7.5), using an ÄKTA purifier at a flow rate of 1 mL min^−1^. Fractions containing target protein as judged by SDS PAGE were pooled. For purification of the periplasmic domain, cells were resuspended in TES buffer (0.2 M Tris pH 8.0, 0.5 mM EDTA, 0.5 M sucrose) at a ratio of 40 mL for a 90 g pellet, stirring gently at 4 °C overnight. After the addition of two volumes of TES/4 buffer (50 mM Tris pH 8.0, 125 mM sucrose, 5 μL of 0.562 mg mL^−1^ benzonase) and stirring for 2 hours, cells were centrifuged (50 000 × *g*, 30 minutes, 4 °C). The supernatant was harvested and diluted with 5 × volume PBS pH 7.4, followed by purification *via* Ni-NTA (but using PBS) and size exclusion chromatography as for the cytoplasmic constructs.

### Protein crystallography

RbdA-PDE (RbdA_549–797_) was concentrated to ∼10 mg mL^−1^ using 10 000 MWCO Vivaspin 2 concentrators (Sartorius). Crystallisation trials were set up in a sitting drop 96-well plate using the Gryphon micro-disperser (Art Robbins Instruments) at 21 °C. Initial crystallisation conditions identified in the Morpheus screen (Molecular Dimensions) were optimised using an Alchemist DT Liquid Handling System (Rigaku), screening buffer system 3 and either precipitant mix 1 or 2 (Molecular Dimensions). The crystal used for structure determination was grown from 100 mM Tris/BICINE pH 9, 34% w/v ethylene glycol/PEG 8000. Data collection was carried out at 100 K on beamline ID23-1 (ESRF). XDS and XSCALE^22,23^ were used for integration and scaling, using zero-dose extrapolation with 0-dose.^24^ Molecular replacement with MOLREP^25^ used PDB:3HV8 as a search model. Iterative model building and refinement were performed with REFMAC5^26^ and COOT.^27^ All other data manipulation was carried out with programs of the CCP4 suite.^28^ The diffraction data are available at https://proteindiffraction.org/project/8pps/ and the final model was deposited with the PDB under accession code 8PPS.

### Enzymatic assay

Reactions were carried out at room temperature in 1 mL reaction buffer (50 mM Bis-Tris Propane buffered at pH 9.35, with 50 mM NaCl, 5 mM MgCl_2_), using a protein concentration of 1.5 μM and 10 μL of 10 mM c-di-GMP (final concentration 100 μM). At the required time intervals, samples of 100 μL were removed, stopping the reaction by the addition of 10 μL of 100 mM CaCl_2_, and placed on ice. A 1 mL Resource-Q column was used on an ÄKTA purifier (GE Healthcare) to separate nucleotides in a 25-column volume linear ammonium bicarbonate gradient (5 mM to 1 M), using a 500 μL injection volume and detection at 253 nm. Standards used were c-di-GMP, pGpG (both Biolog), GTP, and GMP (both Sigma). Calibration curves for c-di-GMP and pGpG used nucleotide concentrations of 0 μM, 5 μM, 10 μM, 20 μM, 40 μM, 60 μM, 80 μM, and 100 μM. Samples were diluted in 890 μL of 5 mM ammonium bicarbonate and run in triplicate. The UNICORN evaluation software was used to integrate elution peaks. Data were fitted in GraphPad Prism (version 7.0). Protocol adapted from ref. 29.

### Bioinformatics, homology modelling, and molecular docking

EMBL SMART^30^ and InterPro^31^ were used for domain annotation, predicting transmembrane regions with TMHMM.^32^ Computed structure models were generated using the Alphafold2 implementation on Google colab v1.5.^33^ Structure comparison and 3D searches used the DALI algorithm.^34^ Identification of protein cavities by the CASTp server (sts.bioe.uic.edu/castp) used a probe radius of 1.4 Å^35^ to determine cavity size and volume. UCSF Chimera was used to determine the solvent-excluded volume based on Connolly's molecular surface calculation.^36^ For docking, the computed model of the periplasmatic domain was converted to PDBQT format using *AutoDockTools* from *MGLTools* version 1.5.7.^37^ The ligand molecules were prepared using *Open Babel* version 3.3.1^38^ and the *prepare_ligand.py* script from the *AutoDockFR* suite.^39^ The docking was carried out with *AutoDock Vina* version 1.2.5^40,41^ using a 13.5 × 15 × 20.25 Å box placed around the largest cavity identified using CASTp.^35^ Rigid side chain docking was used for all ligands.

## Results

### Structure of the dimeric RbdA phosphodiesterase domain

Phosphodiesterases of the EAL type (named after a conserved sequence motif)^11^ are required to dimerise to attain catalytic activity, as was demonstrated for several EAL-type phosphodiesterases.^42,43^ Dimerisation is understood to organise active site formation, enabling metal and substrate binding.^42^ As the previous structure of the PAS–DGC–PDE fragment characterised the auto-inhibited state where the PDE was seen in direct contact with the DGC,^19^ we expressed the autonomous PDE domain to understand whether it is able to fold into an active, dimeric structure.

**Fig. 1:**
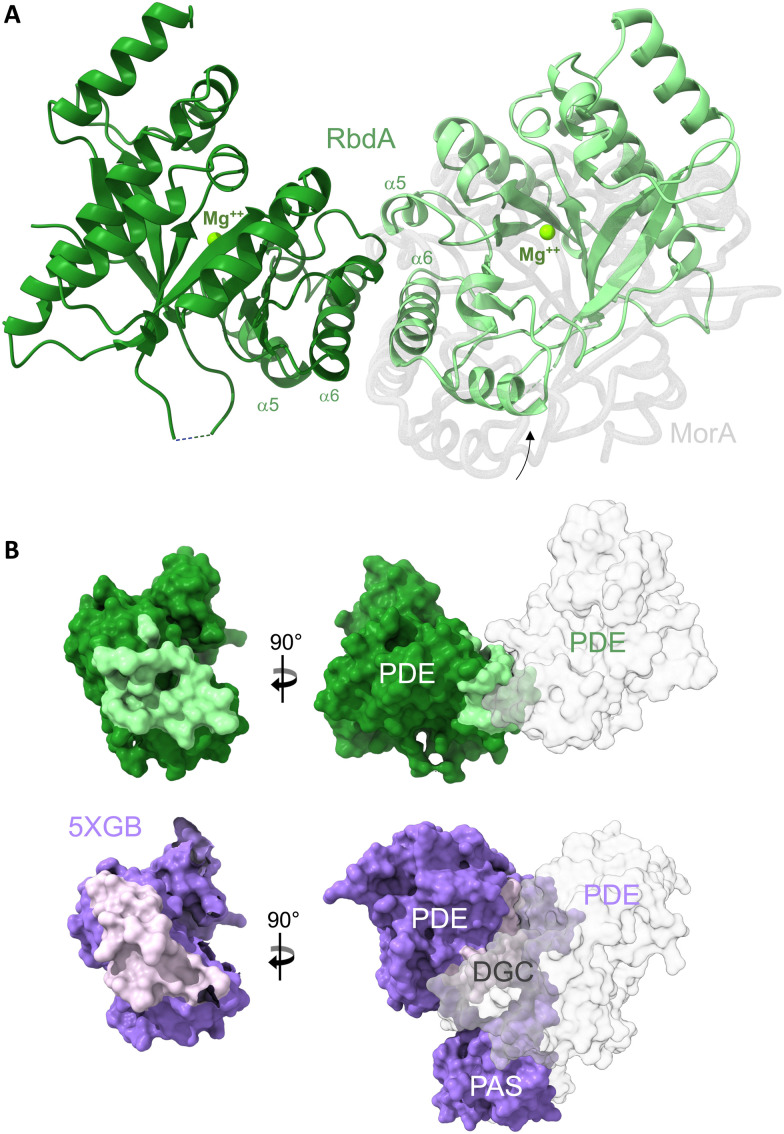
The dimeric structure of the PDE domain from RbdA. (A) The nucleotide-free RbdA PDE dimer has a single magnesium ion bound in each monomer (green sphere) and is shown in cartoon representation (green shades). The dimer interface involves helices α5 and α6. An offset of the RbdA PDE dimer is evident from superposition with the nucleotide-free MorA PDE dimer (PDB:4RNI, offset domain shown in grey). (B) Comparison of interface regions between dimeric PDE and the PDE domain in the PAS–DGC–PDE fragment, formed with PAS and DGC domains (PDB:5XGB).

The structure of RbdA-PDE (RbdA_549–797_) was determined to 2.3 Å resolution, Table 1. RbdA shows an (β/α)_8_-type barrel fold with an inverted first beta-strand. In the structure, RbdA forms a dimer across helices α5 and α6, Fig. 1A. The RbdA-PDE dimer is observed with a single metal and no substrate c-di-GMP or product pGpG bound. Metal binding is identical in both monomers.

Data collection and refinement statistics for RbdA EAL_549–797_. Numbers in parentheses give values for the high-resolution bin

**Table:** 

Data collection	RbdA EAL_549–797_
Diffraction source	ESRF ID23-1
Wavelength (Å)	0.9789
Temperature	100 K
Space group	*P*2_1_2_1_2_1_
Unit cell dimensions	
*a*, *b*, *c* (Å)	64.40, 65.72, 172.25
*α*, *β*, *γ* (°)	90.0, 90.0, 90.0
Resolution (Å)	50–2.3 (2.36–2.3)
*R*_meas_ (%)	5.9 (91.8)
*I*/*σI*	15.90 (1.32)
CC_1/2_	0.999 (0.665)
CC*	0.999 (0.893)
Completeness (%)	93.5 (78.0)
Total reflections	144 478 (5950)
Unique reflections	31 120 (1890)
Multiplicity	4.6 (3.1)
Wilson B (Å^2^)	60.6

**Table:** 

Refinement	8PPS
Resolution (Å)	50–2.3 (2.36–2.3)
No. reflections	29 625 (1802)
*R*_work_/*R*_free_	0.1839/0.2324
No. atoms	
Protein	3997
Ligand/ion	48/2
Water	188
Mean ADP (Å^2^)	62.02
Protein	62.05
Ligand and ion	69.24
Water	59.41
R.m.s deviations	
Bond lengths (Å)	0.0085
Bond angles (°)	1.552
Ramachandran (%, #)	
Preferred	97.82, 489
Allowed	2.22, 11
Disallowed	0.20, 1

Dimer formation is a common feature for EAL-type PDEs, though several different dimers are known.^11,43^ The unifying principle is interaction around helices α5 and α6, where dimerisation leads to changes in the β5–α5 loop structure, which links to substrate and cofactor binding through reorganisation of the active site. The dimer observed here for RbdA is similar to, but slightly offset from, the earlier observed dimeric conformation in the nucleotide-free MorA dimer,^42^Fig. 1A (for comparison with RmcA see Fig. S3, ESI†).

RbdA forms a dimer interface of 715.8 Å^2^, as calculated by PISA.^44^ We compared this interface with interfaces observed in the structure of the autoinhibited state (PDB:5XGB),^19^ obtained with the PAS–DGC–PDE fragment, Fig. 1B. While the PDE show dimer interfaces in both cases, the dimers are different, and the interface is smaller in the autoinhibited state (interface area 434.8 Å^2^). Instead, the PDE interfaces with the PAS domains (interface area 865.0 Å^2^) and the DGC domains (interface area 223.3 Å^2^). These interfaces with PAS/DGC domains in the autoinhibited state overlap with the interface in the dimeric form of RbdA, Fig. 1B, suggesting complex formation is mutually exclusive. We next investigated the consequence of dimer formation for potential PDE activation.

### Dimer formation, metal/substrate binding, and activation

**Fig. 2:**
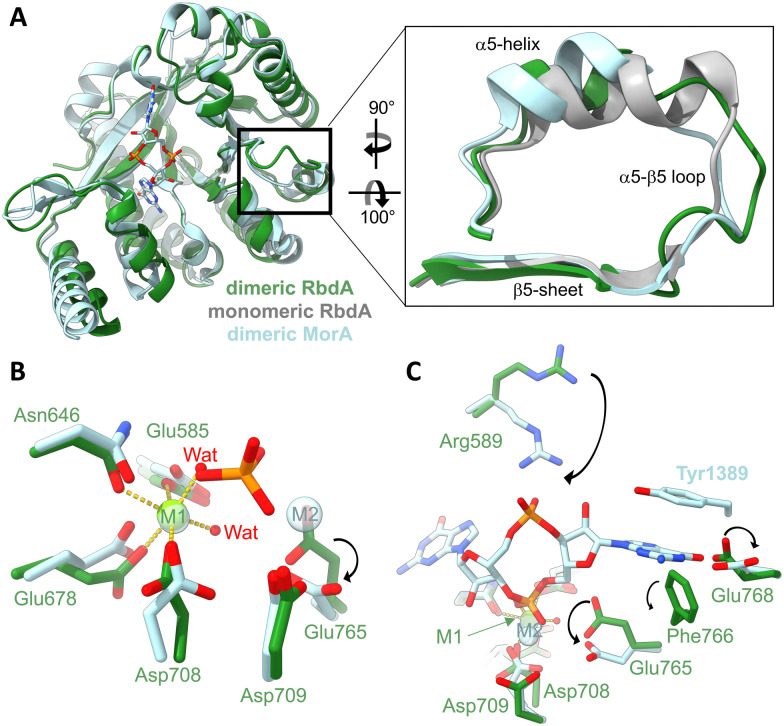
Impact of the dimer formation on active site structure. (A) Superposition of dimeric RbdA-PDE (green) with the structure RbdA-PDE from the PAS–DGC–PDE triple-domain fragment (PDB:5XGE) where the PDE is monomeric (grey). The superposition includes the dimeric substrate-bound structure of MorA (PBD:4RNH^42^). The zoom view shows that helix α5 in dimeric RbdA differs in structure from the autoinhibited monomeric RbdA but is similar to dimeric MorA. (B) Four amino acids and two water molecules coordinate a metal (magnesium) in octahedral geometry in the M1 binding site, similar to the substrate-bound MorA (PBD:4RNH^42^). In substrate bound MorA, the position of one coordinating water is taken by the substrate phosphate (orange/red), and another metal binds to M2, indicated by a different positioning of Asp708 and Glu765. (C) Substrate c-di-GMP binding is characterised by different positioning of Arg589 and Glu768, as seen from comparison of dimeric nucleotide free RbdA and dimeric nucleotide bound MorA. While the aromatic Tyr1389 in MorA is not conserved, Phe766 found in RbdA may perform a similar function and act in substrate pi–pi stacking.

In the dimeric structure, helix α5 is shorter than in the structure of the auto-inhibited PAS–DGC–PDE triple-domain fragment,^19^Fig. 2A. The shorter helix is a hallmark structural change that was previously linked to activation as it allows engagement of the β5–α5 loop with metal cofactors in the active site.^42^

Three metal binding sites have been identified in EAL-type phosphodiesterases.^43^ Nucleotide-free EAL-PDE structures typically contain one metal in what was called the M1 position, and this is also observed for dimeric nucleotide free RbdA, Fig. 2B. Metal coordination in RbdA involves the side chains of Glu585, Asn646, Glu678, Asp708, and two water molecules, leading to an octahedral binding geometry.

In comparison, nucleotide-bound dimeric MorA contains two metal ions,^42^Fig. 2B. Comparison between nucleotide-free RbdA and nucleotide-bound MorA reveals a different positioning of several amino acid side chains to accommodate metal binding. Notably, Asp708 and Glu765 in RbdA would have to take an alternative conformation.^45–47^ Another difference concerns displacement of one of the coordinating waters by the c-di-GMP substrate phosphate.

Further changes in the local structure are required for nucleotide binding, Fig. 2C. These involve Asp708 and Glu765, as well as Arg589, Glu768 side chains. A different orientation of the Phe766 side chain would enable Pi–Pi stacking with the substrate guanine base, akin to the reorientation of Tyr1389 in MorA upon nucleotide binding.^42^

The two aspartate side chains of Asp708 and Asp709 are part of the conserved DDFGTG sequence motif,^42^ which in RbdA is DDFCAG. This sequence segment is part of the β5–α5 loop, which changes structure in response to dimer formation around helices α5 and α6, hence linking dimerisation with metal binding and thus phosphodiesterase activation.

### Influence of the cyclase domain on PDE activity

While the structure analysis presented here suggests that the dimeric form of the PDE domain of RbdA is an activated state, comparison of the interfaces reveals a small interface with the cyclase domain. We therefore wanted to understand whether the DGC can negatively affect PDE activity, and thus employed catalytic analysis together with characterisation of oligomerisation behaviour.

**Fig. 3:**
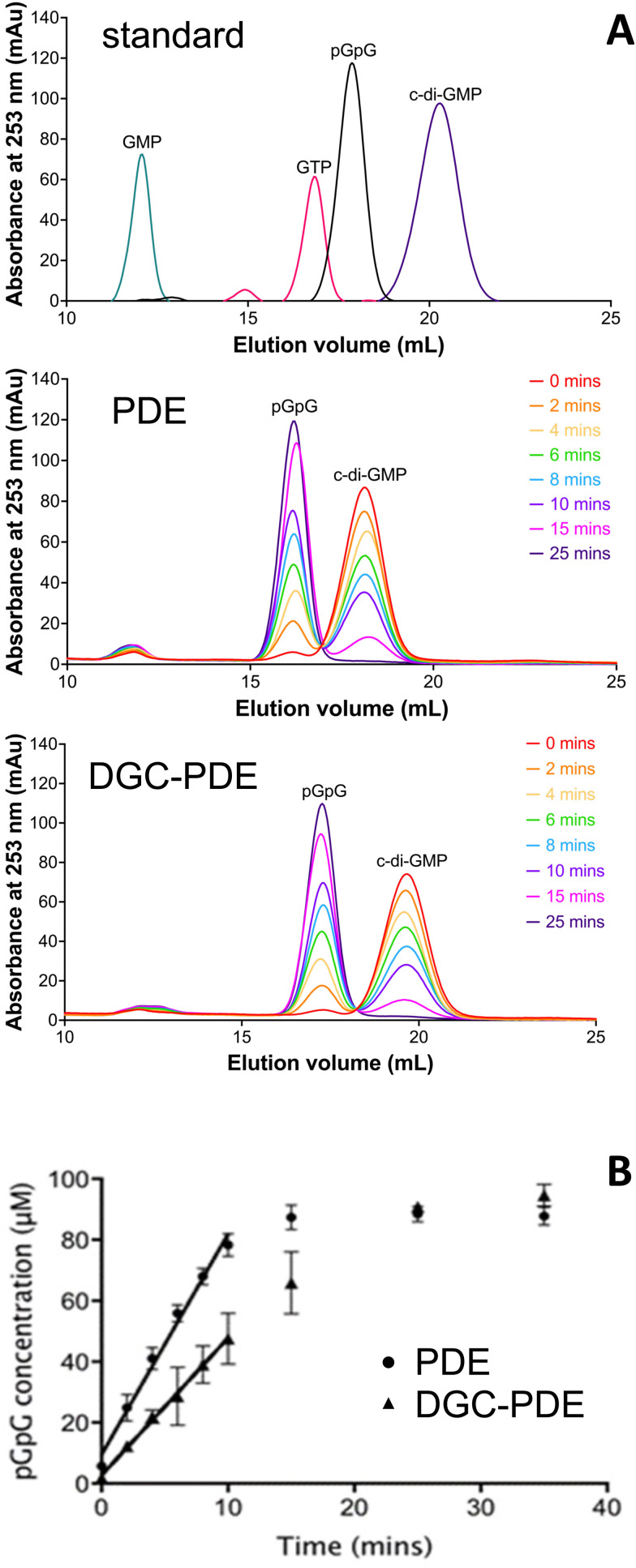
Influence of the cyclase domain on PDE activity. (A) Nucleotide standard (c-di-GMP, pGpG, GTP, and GMP at 100 μM concentration) and enzymatic time profiles using the autonomous PDE (RbdA_549–797_, middle) and the DGC–PDE double domain (RbdA_376–797_, bottom). Samples were separated on a 1 mL Resource Q column and detected by absorbance at 253 nm. (B) Determination of initial rates of phosphodiesterase activity for PDE shown as circles as *y* = 7.24*x* + 9.442 (*R*^2^ = 0.9881) and for DGC–PDE shown as triangles as *y* = 4.507*x* + 2.663 (*R*^2^ = 0.9971); *n* = 3; significance *P* = 0.0002.

We determined the c-di-GMP hydrolytic activity of the isolated PDE domain (RbdA_549–797_) and compared this with the activity determined for the DGC–PDE tandem (RbdA_376–797_). Regression analysis of a time series showed that initial rates for PDE activity are 1.6 times increased for autonomous PDE, compared with DGC–PDE tandem, Fig. 3.

As catalytic activity is controlled by dimerisation, we confirmed the oligomerisation state of the purified proteins in solution. Determination of the apparent molecular weight using a calibrated size exclusion system demonstrated that both the PDE domain and the linked DGC–PDE double domain were mainly dimeric (experimental elution profiles can be found in Fig. S1, ESI†). Together, these data demonstrate that the DGC can exert an inhibitory effect on the catalytic activity of the PDE.

### The periplasmatic domain of RbdA

**Fig. 4:**
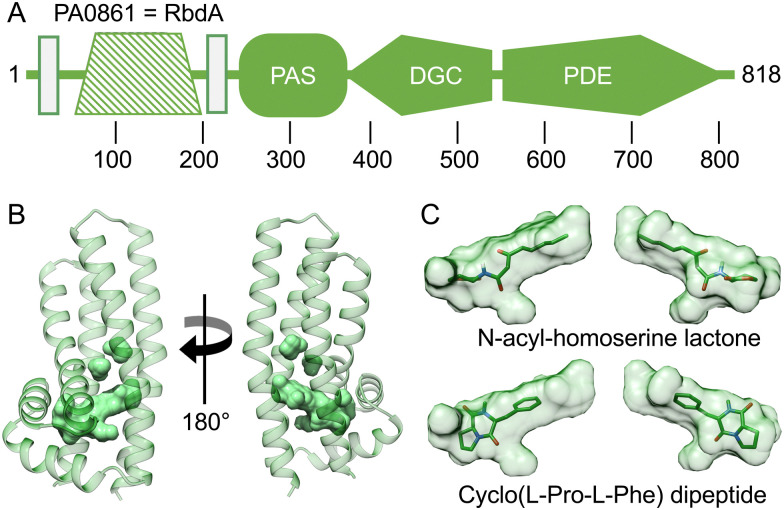
Computed model of the periplasmatic domain. (A) Domain structure as assigned by the EMBL smart server.^30^ Transmembrane segments are shown as rectangular boxes, filled colour indicates availability of crystallographic models, crosshatching indicates the helical domain analysed from computed models. (B) Several cavities were identified using CASTp^35^ (Fig. S2 and Table S1, ESI†). The largest pocket has a molecular surface volume of greater than 400 Å^3^. (C) Molecular docking analysis of *N*-acyl homoserine lactone and the dipeptide Cyclo(l-Pro-l-Phe).

An intriguing feature of RbdA is its N-terminal periplasmatic domain that is interspersed between the two transmembrane segments, Fig. 4A. When investigating domain structure, the periplasmatic domain of RbdA was not recognised by either SMART^30^ or InterPro,^31^ and a functional assignment is lacking.

After confirmation of the transmembrane region with TMHMM,^32^ we selected RbdA_38–201_ for further investigation. The periplasmatic domain RbdA_38–201_ was expressed with a signal sequence for periplasmic localisation. Despite multiple attempts, we were unable to crystallise the periplasmic domain of RbdA.

Instead, we used AlphaFold2 (AF2/colab implementation^33^) to compute a structural model, Fig. 4B. We then analysed this model for ligand binding pockets using CASTp.^35^ The analysis identified a large cavity without openings to the environment inside the periplasmatic domain, lined mainly by small aliphatic residues (analysed in Table S1 and Fig. S2, ESI†).

To gain further functional insight, we submitted the computed model to a search for related structures, using DALI.^34^ While no structure was found that would match the entirely helical fold similarities with alpha-helical bundle proteins were evident. In particular, DALI identified periplasmatic carbohydrate sensor domains of histidine kinases. In the LytS protein (PDB:5XSJ), a similar domain can influence oligomerisation behaviour on d-xylose binding.^48^ Further similarities exist to chemoreceptors in chemotaxis pathways. These include the sensor domains of histidine kinases (PDB:4K0D^49^) or domains able to adapt to specific chemo-attractants such as malate, succinate, or acetate (PDB:2YFB^50^) or the *Pseudomonas putida* receptor PcaY that was characterised in complex with quinate (PDB:6S38^51^). Analysis of the size of the binding pocket may suggest hydrophobic ligands to a size of ∼600 Da. Potential ligands include acyl-homoserine-lactones (AHL), which are known to be involved in quorum sensing.^52^ Molecular docking showed that the AHL *N*-3-oxo-octanoyl-l-homoserine lactone did fit the C8 carbon tail well into the pocket density, giving a calculated affinity of −8.03 kcal mol^−1^, Fig. 4C. We also investigated amino acid ligands such as the dispersal signal l-glutamate^21^ and cyclic dipeptides that have anti-biofilm activity.^53^ The dipeptide Cyclo(l-Pro-l-Phe) gave a good fit to the pocket with a calculated affinity of −6.64 kcal mol^−1^, Fig. 4C.

## Discussion

Due to its ability to form biofilms, *P. aeruginosa* has been shown to evade the host's inflammatory response and antibiotic therapies.^54^ Thus, mechanisms for dispersal are intensely investigated. As the biofilm lifestyle is linked to high intracellular concentration of the secondary messenger c-di-GMP, study of phosphodiesterases (PDEs) involved in degradation and turnover of the nucleotide messenger are opportune.

As PDEs are typically found in multi-domain proteins, it is important to gain insight into the different structural states that are able to regulate enzymatic activity. A specific focus is to understand how the opposing activities of PDE and cyclase domains are balanced.

The EAL type PDE is well understood from structural studies, and together these studies link substrate and metal binding with dimerisation and activation.^29,42,43,55^ As RbdA overall negatively regulates *P. aeruginosa* biofilm formation, it must be an active PDE under physiological conditions.^18^ However, the structure of the PAS–DGC–PDE fragment shows that both the PDE and DGC are autoinhibited due to the substrate binding sites of the PDE and the DGC facing each other.^19^

The structure of the dimeric RbdA PDE presented here is primed for substrate binding. Thus, while activation is suppressed in the multi-domain arrangement seen in the autoinhibited state, the isolated PDE has the propensity to dimerise and become activated. Whilst we do not know the activated dimer in the context of the full-length structure, the analysis presented in Fig. 1B shows that interfaces of the PDE are mutually exclusive in active and autoinhibited conformation. Indeed, interface areas required for the activated state overlap with areas that are involved in interactions with PAS and DGC domains in the autoinhibited state.

We tested the activation model and present biochemical data that show that the linked DGC–PDE is less active than the PDE alone. While dimerisation is also required for the DGC domain to activate c-di-GMP synthesis, bringing two DGC domains each carrying a GTP substrate together, the DGC on its own is mainly monomeric while the PDE and DGC–PDE are both dimeric (Fig. S1, ESI†). Our data establish the principle that the DGC in RbdA can directly exert an inhibitory effect over the PDE.

It has been reported that presence of GTP or the non-hydrolysable GTP analogue Guanosine 5′-β-γ-imido triphosphate (GMPPNP) enhance PDE activity of RbdA. Notably, stimulation by these nucleotides depended on the presence of an intact DGC domain,^19,20^ which has also been described in other *P. aeruginosa* PDE proteins such as RmcA (PA0575, compare Fig. S3, ESI†)^55,56^ and in the mycobacterial protein DcpA.^57^ Nucleotide binding to the A-site of the DGC might promote this activation^19,58^ by releasing the autoinhibited conformation and supporting structural changes that are required for PDE dimerisation.^19,42,58^

Our computational model of the RbdA periplasmatic domain reveals a unique helical fold with a large binding pocket. We have tested different functional hypotheses, investigated the size of the pocket, and explored structural similarities to other (periplasmatic) protein domains. The suggestion that the domain may be a glutamate sensor is plausible since RbdA acts as a sensor to such environmental triggers, as shown earlier;^21^ however the binding pocket is much larger than glutamate, Fig. 4C. A well-characterised molecule for quorum sensing and biofilm control, *N*-acyl-homoserine lactone,^59^ equally is too small to fill the entire pocket. The pocket is large enough to bind a hydrophobic ligand with a size of 600 Da or larger, as seen by an overlay with Cyclo(l-Pro-l-Phe), but the physiological ligand is presently unknown.

While the multiple layers of control of PDE function are critical for understanding phosphodiesterase function and bacterial lifestyle, they may also provide future avenues to be explored in the search of biofilm dispersing agents. In *Pseudomonas aeruginosa*, 41 proteins with either diguanylate cyclase (DGC) or phosphodiesterase (PDE) domains were identified,^12^ several of these sharing similar architecture with respect to domain composition. All these proteins typically contain N-terminal domains that are attributed to regulatory functions, while the DGC/PDE domains are the C-terminal output domains. Understanding the intricacies of multi-domain structure might lead to insights into functional diversification during evolution.

## Author contributions

JSW, IT, and MAW designed the study. CC and JC conducted experimental work. CC, JC, MM, KB, and IT performed data analysis. CC, JC, and IT drafted the manuscript. CC, MM, KB, and IT revised the manuscript.

## Data availability

Data for this article, including raw enzymatic data, AlphaFold2 structures, and pocket analysis are available at the University of Southampton repository system, Pure, at https://doi.org/10.5258/SOTON/D3183. Crystallographic data for the EAL domain of RbdA has been deposited at the PDB under 8PPS and can be obtained from https://doi.org/10.2210/pdb8PPS/pdb and diffraction images can be obtained from https://doi.org/10.18430/M38PPS.

## Conflicts of interest

There are no conflicts to declare.

## Supplementary Material

CB-OLF-D4CB00113C-s001
