# Supplementary material for: Control of phosphodiesterase activity in the regulator of biofilm dispersal RbdA from Pseudomonas aeruginosa
Source: RSC Chem Biol. 2024 Aug 27;5(10):1052–9. doi: 10.1039/d4cb00113c (PMC11372557; doi:10.1039/d4cb00113c)
Supplement: CB-005-D4CB00113C-s001 [file CB-005-D4CB00113C-s001.pdf]

Supplementary Information (SI) for RSC Chemical Biology.
This journal is © The Royal Society of Chemistry 2024

Supplement to:

Control of phosphodiesterase activity in the regulator of biofilm dispersal RbdA from *Pseudomonas aeruginosa*

Charlotte Cordery[a,b,c,d], Jack Craddock[a,b], Martin Malý[a], Kieran Basavaraja[a,b,c,d] Jeremy S. Webb[a,b], Martin A. Walsh[c,d] and Ivo Tews[a,b]

[a] Biological Sciences, Institute for Life Sciences, University of Southampton, Southampton, SO17 1BJ, UK

[b] National Biofilms Innovation Centre, University of Southampton, Southampton, SO17 1BJ, UK

[c] Diamond Light Source, Harwell Science and Innovation Campus, Didcot, Oxfordshire, OX11 0DE, UK

[d] Research Complex at Harwell, Harwell Science and Innovation Campus, Didcot, Oxfordshire, OX11 0FA, UK

Address correspondence to Ivo Tews, ivo.tews@soton.ac.uk

This supplement contains:
Supplementary Table S1
Supplementary figures S1+S2

Colour	MS volume (Å^3)	SA volume (Å^3)	Pocket MS area (Å^3)	Pocket SA area (Å^3)	Openings #	lining amino acids
green	430.8	56.2	403.6	144.1	0	Ser5, Tyr6, Gly9, Glu10, Trp13, Asp49, Met52, Ile70, Asn75, Ile83, **Phe96**, Leu102, **Trp103**, Gly106, Val148, Ala151, Ala152, **Phe155**
green	13.2	0.0	27.1	0.0	0	**Phe96**, Ile100, **Trp103,** **Phe155**
blue	35.3	0.2	59.1	3.2	0	Trp13, Pro46, Arg50, Asp107, **Val110**
grey	22.5	0.1	39.4	1.1	0	Gly9, Leu12, **Asp49**, Gly73, Asn75
purple	51.2	1.2	72.0	8.4	0	Gln17, Tyr109, **Val110**, Leu113, Ile144, **Val148**

Table S1. Analysis of cavities in the RbdA periplasmatic domain RbdA$_{38-201}$ (computed model, AF2). Color code relates to amino acids lining the cavity as seen in Figure S2. The largest pocket volume is explored in Figure 4.

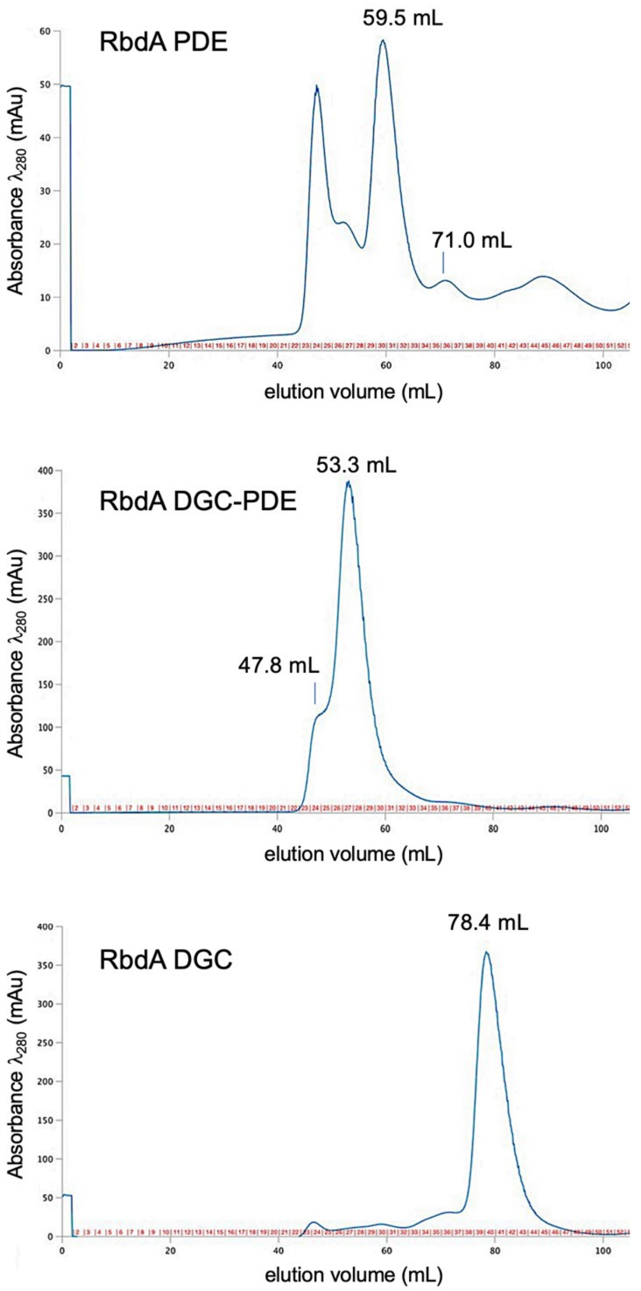

Figure S1. Experimental size determination of RbdA constructs. The PDE domain (RbdA$_{549-797}$) mainly eluted as a dimer at approximately 59.5 mL, with a small peak present for the monomer at approximately 71 mL. The linked DGC-EAL construct (RbdA$_{376-797}$) eluted as a dimer at approximately 53.3 mL. The autonomous DGC domain was (mainly) monomeric (RbdA$_{376-536}$) and eluted at approximately 78.4 mL. Determined using a HiLoad 16/600 Superdex 75 prep grade column.

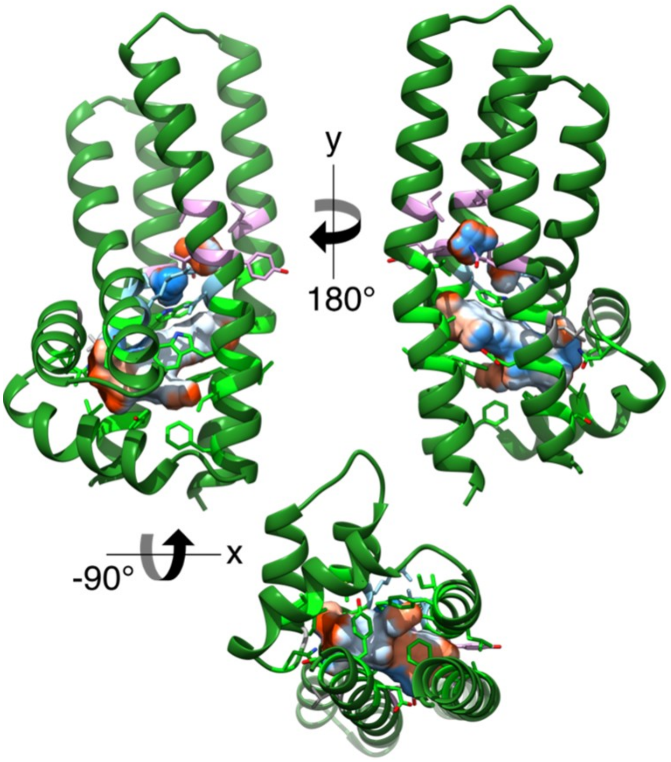

Figure S2. Computed model of RbdA$_{38-201}$ periplasmatic domain. The colors indicate amino acids lining different pockets as given in Table S1. Selected amino acid side chains lining the pockets are shown as sticks.

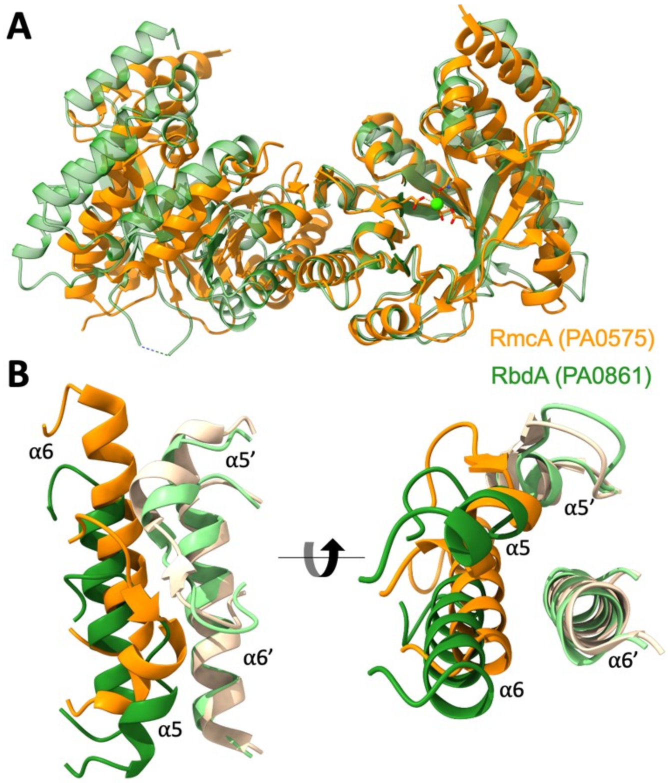

Figure S3. Superposition of of RbdA (green, translucent) and RmcA (orange, solid; 5M3C[54]). A, the dimerisation interface, cartoon representation; compare Figure 1. **B,** helices $\alpha5$ and $\alpha6$ from the interface in RmcA (orange and light orange) with a small β-sheet, as opposed to RbdA (dark and light green).
